# Uncertainty in the spatial distribution of tropical forest biomass: a comparison of pan-tropical maps

**DOI:** 10.1186/1750-0680-8-10

**Published:** 2013-10-26

**Authors:** Edward TA Mitchard, Sassan S Saatchi, Alessandro Baccini, Gregory P Asner, Scott J Goetz, Nancy L Harris, Sandra Brown

**Affiliations:** 1 School of GeoSciences, University of Edinburgh, Crew Building, The King’s Buildings, Edinburgh EH9 3JN, UK; 2 Jet Propulsion Laboratory, California Institute of Technology, Pasadena, CA 91109, USA; 3 Woods Hole Research Center, 149 Woods Hole Road, Falmouth, MA 02540-1644, USA; 4 Department of Global Ecology, Carnegie Institution for Science, 260 Panama Street, Stanford, CA 94305, USA; 5 Ecosystem Services Unit, Winrock International, 2121 Crystal Drive, Suite 500, Arlington, VA 22202, USA

**Keywords:** Aboveground biomass, Carbon, Data inter-comparison, *Maxent*, *Random forest*, REDD, REDD+, Remote sensing, Tropical forests, UNFCCC

## Abstract

**Background:**

Mapping the aboveground biomass of tropical forests is essential both for implementing conservation policy and reducing uncertainties in the global carbon cycle. Two medium resolution (500 m – 1000 m) pantropical maps of vegetation biomass have been recently published, and have been widely used by sub-national and national-level activities in relation to Reducing Emissions from Deforestation and forest Degradation (REDD+). Both maps use similar input data layers, and are driven by the same spaceborne LiDAR dataset providing systematic forest height and canopy structure estimates, but use different ground datasets for calibration and different spatial modelling methodologies. Here, we compare these two maps to each other, to the FAO’s Forest Resource Assessment (FRA) 2010 country-level data, and to a high resolution (100 m) biomass map generated for a portion of the Colombian Amazon.

**Results:**

We find substantial differences between the two maps, in particular in central Amazonia, the Congo basin, the south of Papua New Guinea, the Miombo woodlands of Africa, and the dry forests and savannas of South America. There is little consistency in the direction of the difference. However, when the maps are aggregated to the country or biome scale there is greater agreement, with differences cancelling out to a certain extent. When comparing country level biomass stocks, the two maps agree with each other to a much greater extent than to the FRA 2010 estimates. In the Colombian Amazon, both pantropical maps estimate higher biomass than the independent high resolution map, but show a similar spatial distribution of this biomass.

**Conclusions:**

Biomass mapping has progressed enormously over the past decade, to the stage where we can produce globally consistent maps of aboveground biomass. We show that there are still large uncertainties in these maps, in particular in areas with little field data. However, when used at a regional scale, different maps appear to converge, suggesting we can provide reasonable stock estimates when aggregated over large regions. Therefore we believe the largest uncertainties for REDD+ activities relate to the spatial distribution of biomass and to the spatial pattern of forest cover change, rather than to total globally or nationally summed carbon density.

## Background

The clearing of tropical forests and their conversion to other land uses has resulted in gross emissions of 0.45 – 1.7 Pg C year^-1^ (90% prediction interval) from 2000–2007, equivalent to 5-19% of global anthropogenic CO_2_ emissions [1-3]. Intact tropical forests are, however, thought to be serving as a carbon sink of similar magnitude, capturing an estimated 0.55-1.49 Pg C year^-1^, equivalent to 6-17% of anthropogenic CO_2_ emissions, over the same period [2]. While there are many other reasons to protect tropical forests, the preservation of their carbon stocks and their potential as a future carbon sink has motivated a policy priority among the international community for their protection in order to reduce greenhouse gas (GHG) emissions, with associated benefits to society provided by their ecosystem services [4].

Many different schemes have been pursued to conserve tropical forests, but all rely on the quantification of stored carbon stocks to allow a calculation of avoided GHG emissions. The UN Framework Convention on Climate Change (UNFCCC) initiative “Reducing Emissions from Deforestation and forest Degradation” (REDD+, [5]) may create both social and economic incentives for conservation of forests in tropical countries. At an international level, REDD+ remains in negotiation within the UNFCCC, with the goal to include REDD+ in the next global climate change agreement. However, pilot and preparatory activities are already occurring at a national level, largely funded by UN-REDD (a consortium of the FAO, UN and UNEP), the Forest Carbon Partnership Facility (World Bank), and individual governments, especially Norway [6]. Parallel to the main REDD+ process, Norway has set up bilateral deals with Brazil, Guyana, Indonesia and Tanzania that allow for the transfer of up to US$1 billion for conservation and development, in return for the countries meeting targets for reducing deforestation rates [7]. Furthermore there are already many voluntary REDD+ projects, generating credits primarily under the Verified Carbon Standard (VCS), with total REDD+ credit sales equal to $85 million in 2010 [8]. These projects are increasing in number, meaning that there is already some implementation of REDD+ in many tropical forest regions.

Under the UNFCCC, countries planning to participate in the REDD+ mechanisms are required to use the Intergovernmental Panel on Climate Change (IPCC) GHG accounting framework for estimating their anthropogenic emissions caused by deforestation and forest degradation [9]. One of the key inputs into the IPCC framework is the carbon stocks of the forests undergoing change. The difference between the pre- and post- deforestation or degradation carbon stocks is the 'emission factor’, which is the carbon emissions per unit area due to forest cover change. The product of the emission factor and the area of forest change provides the estimate of the total carbon emissions.

Countries participating in a future UNFCCC agreement will likely need to assess and monitor their carbon stocks regardless of their inclusion in REDD+. One approach often followed to obtain carbon stock estimates is to map vegetation types within a landscape and assign a carbon density value to each vegetation type, using either international or locally-derived values from field-based inventory [9]. However this method can have high uncertainty, especially over large areas or when using generic carbon density values, so to maximise potential financial benefits countries may opt to produce spatial maps of their biomass stocks, using field-calibrated remote sensing observations. No current satellite can directly estimate aboveground biomass (AGB), so proxies related to forest canopy colour, seasonality parameters, elevation, or the canopy structure are used to estimate and spatially model AGB [10-14].

Two recent maps have been published using this approach to estimate biomass across the tropics at a 1 km resolution [15], subsequently described as 'RS1’, and a 463 m resolution [16], 'RS2’. These resolutions are considered high enough to be used by carbon forestry projects [9]. Both maps use spaceborne LiDAR data from the Geoscience Laser Altimeter System (GLAS) as samples of forest structure distributed across the tropics, but the two approaches use a different method to extend the isolated GLAS footprints to full-coverage AGB maps. The differences can be summarized as follows:

i) **GLAS datasets:** Both studies independently downloaded, processed and filtered the GLAS dataset for cloud and slope effects and other potential artefacts. In *RS1*, filters were introduced to remove all GLAS shots over slopes > 20% and ground elevations with > 100 m difference from a global digital elevation model, the Shuttle Radar Topography Mission (SRTM) data at 90 m resolution; in *RS2*, the filter removed all GLAS shots that differed from SRTM elevation by > 25 m. In both cases this was done because forest height estimates over sloped terrains may have large biases, causing overestimation of the estimated tree height. Both methods included a series of filters based on the shape of the waveform and the signal-to-noise ratio.

ii) **Estimating AGB from GLAS using field plots:** Field plots are used to convert millions of individual LiDAR waveforms collected by the GLAS sensor with an approximately 65 m footprint into AGB estimates. *RS1* uses a two-stage process, first building a model to predict Lorey’s height (basal-area weighted height) from the LiDAR waveforms using 295 field plots located under GLAS footprints in South America [17], and then deriving three separate continental equations relating Lorey’s height to AGB using a set of 493 field plots [15]. The AGB values for the field plots are derived from the 3-parameter tropical forest allometric equations including tree diameter, wood density, and height from [18]. The field plots were distributed over three continents, had sizes ranging from 0.2 to 1.0 ha, with the majority of plots being at least 0.25 ha, and included all trees > 10 cm in diameter measured above buttresses.

*RS2* instead builds a model directly relating GLAS waveform characteristics to AGB from 283 calibration field plots located under GLAS footprints [16]. The plots are 40 m × 40 m (0.16 ha) in size and include all trees > 5 cm in DBH. Unlike *RS1*, in *RS2*, the field data are converted to AGB using allometric equations without tree height from the same study [18]: *RS1* uses the 3-parameter equation, whereas *RS2* uses the 2-parameter equation, including diameter and wood density but excluding height.

The conversion of the GLAS data to AGB in both approaches ignores the potential variations of forest wood density over the landscape and at regional scales: while biomass estimation of the plot data for both maps was based on equations that included wood density as one of the independent variables, the functions that related the GLAS data to the plot-based biomass estimates did not include any parameter to reflect the spatial variability of wood density.

iii) ** Creation of training and test datasets from GLAS:** For *RS1,* GLAS AGB estimates are only used in creating the map if at least 5 LiDAR footprints fall within the same 1 km pixel; this gave 160,918 pixels (with the AGB estimate for each the average of at least 5 LiDAR footprints) for use in training and testing the AGB prediction model. For *RS2* GLAS AGB estimates were used if more than 5 footprints were located in a 463 m pixel for America and Africa, and 3 or more for Asia, giving 58,476 pixels available for training and testing.

iv) **Additional training dataset from field plots:** Additionally for *RS1* 4,079 field plots were included in the model although, as these were clustered, they were averaged if multiple plots occurred within the same 1 km pixel, reducing the total to 1,877 pixels. No field dataset was used directly for training or testing of *RS2.*

v) **Creating continuous AGB maps:** The point AGB estimates were averaged to give single AGB estimates at the pixel level, then extrapolated across the full pantropics using visible- and infra-red spectrum optical data from the Moderate Resolution Imaging Spectroradiometer (MODIS) sensors, elevation data from SRTM, and in the case of *RS1,* QUIKSCAT scatterometer data. The precise MODIS data layers used and cloud filtering applied differ considerably between the studies, with *RS1* using Leaf Area Index (LAI) and the Normalised Difference Vegetation Index (NDVI), and *RS2* using all the land bands excluding the blue band from the Nadir Bidirectional Reflectance Distribution Function-Adjusted Reflectance (BRDF), the Enhanced Vegetation Index (EVI2), the Normalized Difference Infrared Index (NDII2), and the MODIS Land Surface Temperature products. The extrapolation of biomass is performed using non-linear, non-parametric models, *Maxent* in *RS1* and *Random Forest* in *RS2*, with in both cases a percentage of input data held back for testing (40% for *RS1*, 10% for *RS2*).

vi) **Uncertainty estimates:***RS1* additionally produced a spatial uncertainty map, giving an error estimate for every pixel, through bootstrapping the input ground and LiDAR datasets and propagating errors through the model. *RS2* estimated uncertainty at the dataset and country level using a Monte Carlo approach.

Here we present a detailed comparison of the outputs of both maps, both directly at the pixel level, and in aggregate over different landcover type classes and countries. However, while comparisons between the maps are interesting, they are of limited use in either confirming the validity of the mapping approach, or stating whether one map should be used preferentially to the other. We cannot use comparisons to field plots to provide these assessments for two reasons: first, the vast majority of well-geolocated recent scientific field plots known to the authors were used in one or other of the maps; and second, all field plots are very much smaller than the pixel size of the maps, and thus only useful in showing if there is large divergence between the maps and ground data, not in providing a quantitative accuracy assessment [19]. We therefore compare the maps to two entirely independent, large-scale ancillary AGB datasets: the country biomass stocks from the FAO Forest Resource Assessment (FRA) estimates [20], and a high resolution (100 m) LiDAR-derived map for a 16.5 million hectare region of the Colombian Amazon (*RS3*) [21].

## Results and discussion

### Direct comparison of the pantropical biomass maps

**Figure 1:**
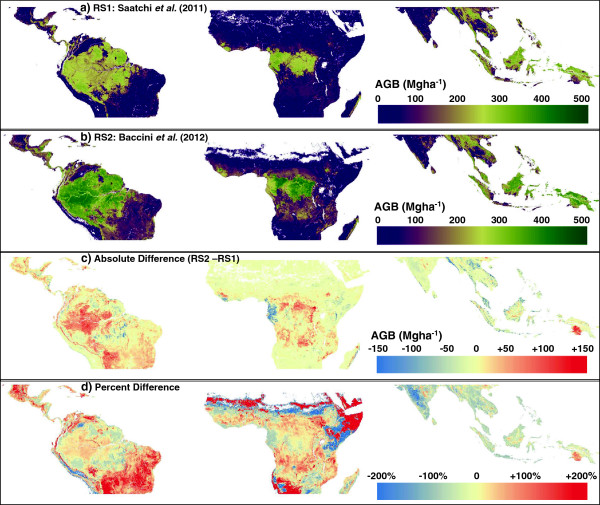
**A comparison of two remote sensing based maps of aboveground biomass (AGB) across the tropics: a) AGB for *RS1 ***[15]**; b) AGB for *RS2 ***[16]**; c) absolute difference, *RS2 *- *RS1*; d) the percent difference between *RS2 *and *RS1*.** The projection is sinusoidal, an equal area projection.

Summing the *RS1* and *RS2* maps by continent gives similar mean and total values (Table 1), with the *RS1* carbon stock estimates across the tropics about 10% lower than *RS2*, driven mostly by an 18% difference in tropical Latin America*.* However, much more dramatic differences are seen when the two maps are compared visually (Figure 1). Absolute differences are most pronounced over tropical forest areas: *RS1* estimates are considerably lower in the central and western Amazon, central and eastern Congo basin, and southern Papua New Guinea, whereas conversely *RS2* has lower estimates in the south-eastern Amazon, the western Congo basin, and parts of South-East Asia. Large differences are also visible over woodland and savanna vegetation, but with more consistency: in general *RS2* estimates are higher than *RS1* in mid- and low- biomass vegetation (with some exceptions, e.g. Kenya and Ethiopia).

**Table 1:** Mean and total biomass stocks by continent

**Continent**	***RS1*** [15]		***RS2*** [16]		**Area compared**
	**Mean**	**Total**	**Mean**	**Total**	**(km**^**2**^**)**
	**(Mg ha**^**-1**^**)**	**(PgC)**	**(Mg ha**^**-1**^**)**	**(PgC)**	
Africa	50.8	56.2	58.4	64.5	22,105,436
Americas	129.8	95.5	158.1	116.3	14,713,658
Asia	160.2	51.7	144.9	46.8	6,457,241
**Pan-tropics**	**94.0**	**203.4**	**105.2**	**227.6**	**43,276,334 km**^**2**^
	**Mg ha**^**-1**^	**PgC**	**Mg ha**^**-1**^	**PgC**	

Comparison of the mean and total aboveground biomass (AGB) stocks estimated for continental regions covered by the two maps. See Figure 1 for areas compared: these do not include whole continents but only extend from the Tropic of Cancer to the Tropic of Capricorn. Many areas mapped in *RS1* (including Australia, southern Latin American and southern Africa) are not included in *RS2*, and are thus excluded from the above table. Water bodies are excluded.

**Figure 2:**
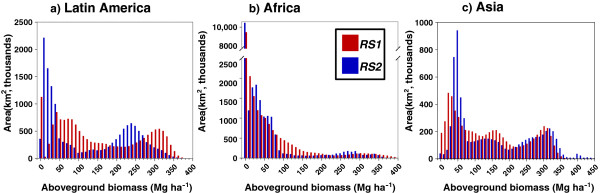
**Histograms showing aboveground biomass (AGB) distributions of *RS1 *** **and *RS2*, in 10 Mg ha**^**-1**^**bins, for the tropical regions of a) Latin America; b) Africa; and c) Asia.**

Comparing histograms of the biomass distributions shows that the differences are not consistent between continents (Figure 2). In Latin America both *RS1* and *RS2* have clear bimodal distributions, but the distributions differ markedly between the two datasets. Both peaks are offset to lower values for *RS1* compared to *RS2*, with the savanna (cerrado) peak dominated by values between 10 and 50 Mg ha^-1^ in *RS1* and 30–100 Mg ha^-1^ in *RS2*, and the tropical forest peak centred around 240 Mg ha^-1^ in *RS1* and 310 Mg ha^-1^ in *RS2.* The distributions for Africa are closer to negative-J distributions, with the dominance of grassland and savanna resulting in a much higher frequency of low biomass classes than high biomass classes. However the differences between *RS1* and *RS2* in Africa are consistent with those in Latin America: once again there is bimodality, and in both cases the peaks are shifted to the left in *RS1* compared to *RS2.* The rainforest peaks are more similar to each other in Africa than in South America, with the clearest difference being the much higher frequency of 90 to 170 Mg ha^-1^ in *RS2* than *RS1.* The picture is different again in Asia, with biomass appearing to be trimodally distributed in both datasets. In Asia, in contrast to the others, there is evidence that the lowest biomass peak is shifted towards higher biomass values in *RS1* compared to *RS2*, though it may be that this peak occupies a wider range in *RS2*; the intermediate peak has higher values in *RS2* than *RS1* throughout; and the high biomass peak has a similar shape and position in both distributions.

#### Comparison by vegetation class

**Figure 3:**
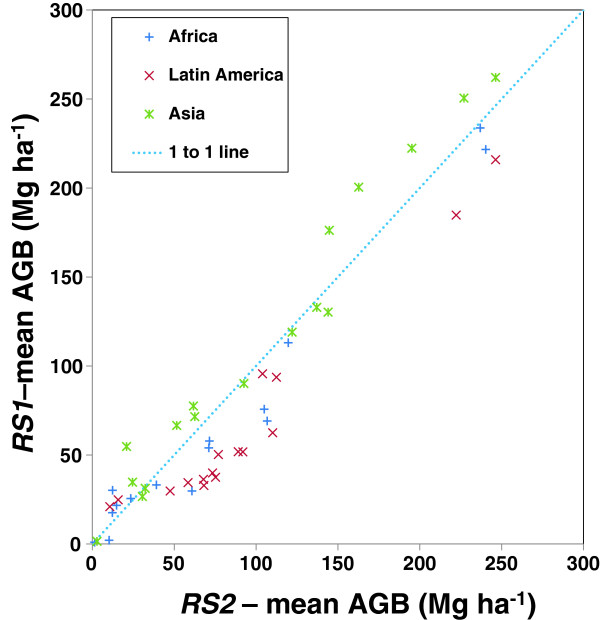
**Mean aboveground biomass (AGB) of vegetation classes from the Global Land Cover 2000 dataset **[34]**, split by continents, between *RS1 *and *RS2 *.** The full data are included in Additional file 1: Table S1.

Subsetting the biomass distribution using a vegetation map shows that differences are not consistent among classes or continents (Figure 3, Additional file 1: Table S1). There are no large outliers, with no points particularly far from the 1:1 line, but in general again *RS1* <*RS2* in Africa and Latin America, and *RS1 > RS2* in Asia. Looking across the dataset the largest absolute differences are in the “Deciduous broadleaved closed forest”, “Needle-leaved evergreen forest”, “Regularly flooded shrub” and “Closed-open evergreen shrub” classes, all of which differ by greater than 34 Mg ha^-1^. Some important classes, for example “Broadleaved evergreen forest”, differ in the sign of their difference between continents: *RS1* is smaller than *RS2* by 18.7 Mg ha^-1^ and 30.4 Mg ha^-1^ in Africa and Latin America respectively, but greater in Asia by 15.8 Mg ha^-1^. This is a relatively consistent pattern, with 5 of 15 classes having *RS1* <*RS2* in African and Latin America, but *RS2* >*RS1* in Asia.

We find no obvious link between the different spatial distribution of field training plots used in the two datasets (which are mostly located in intact tropical forest, with some located in tropical savanna woodland) and the degree of difference between the corresponding vegetation classes. For example there is a large difference in the class best sampled in both datasets (“Tree cover, broadleaved, evergreen”), and a comparatively small difference for “Tree Cover, regularly flooded, saline water”, a class which was not included in the LiDAR calibration datasets of either map, and that is known to have a distinct vegetation structure.

#### Comparison by country total

**Figure 4:**
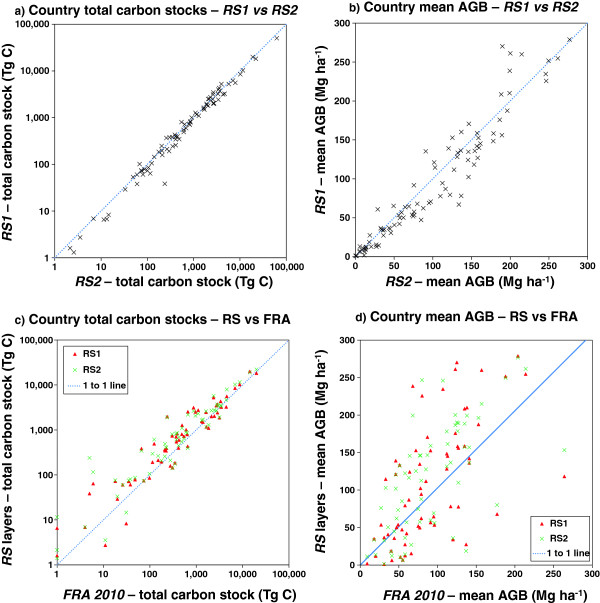
**A comparison of carbon stocks and mean aboveground biomass (AGB) values by country for *RS1*, *RS2 *** **and the FAO Forest Resource Assessment (FRA) 2010: a) Country total carbon stocks – *RS1 *** **vs *RS2*; b) Country mean AGB – *RS1 *** **vs *RS2*; c) Country total carbon stocks – *RS1 & RS2 *** **vs FRA; d) Country mean AGB – *RS1 & RS2 *** **vs FRA.**

Comparisons at a country level show much greater levels of agreement between the maps (Figure 4a-b, Additional file 2: Table S2). In terms of the total biomass for a country, convergence is expected as the area term is identical across both maps. However, more surprisingly, there is also a good deal of convergence in mean AGB across countries. In both cases performing Reduced Major Axis regressions (appropriate as the errors should be equally distributed on both axes) produced best fit lines that were significantly different from the 1:1 line at the 95% confidence level, with 95% confidence intervals for slopes ranging from 0.88-0.94 for country stocks, and 0.96-0.99 for mean biomass, suggesting *RS1* does on average predict significantly lower AGB than *RS2.* However, the r-squared values for the RMA regression lines were 0.97 for total country stocks and 0.91 for mean values, suggesting that there is a strong positive relationship between the datasets. There were some significant outlier countries however, for example Haiti, Gambia and Botswana were estimated as containing 80%, 76% and 60% more carbon using *RS2* than *RS1*, whereas by contrast East Timor, Kenya and Equatorial Guinea are estimated as containing 49%, 47% and 42% more biomass in *RS1* than *RS2* (Additional file 2: Table S2). Another way to look at this dataset is to calculate the Root Mean Squared Error (RMSE) in mean carbon stocks between the countries; this value is 23.1 Mg ha^-1^ when comparing *RS1* and *RS2* for the 92 countries (Table 2).

**Table 2:** Comparison of mean biomass stocks and RMSE values for countries by continent

**Continent**	**Mean country AGB**			**RMSE for country mean AGB**			**Number countries compared**
	***RS1***	***RS2***	**FRA2010**	***RS1*** **vs** ***RS2***	***RS1*** **vs FRA2010**	***RS2*** **vs FRA2010**	
	**(Mg ha**^**-1**^**)**	**(Mg ha**^**-1**^**)**	**(Mg ha**^**-1**^**)**	**(Mg ha**^**-1**^**)**	**(Mg ha**^**-1**^**)**	**(Mg ha**^**-1**^**)**	
Africa	68.9	74.2	83.4	25.0	54.8	44.3	37
Americas	113.8	130.2	99.0	20.8	50.4	63.5	28
Asia	157.1	142.2	99.0	27.5	102.4	85.7	14
**Pantropics**	**100.5**	**106.1**	**91.0**	**24.1**	**65.0**	**59.7**	**79**

Comparison of mean aboveground biomass (AGB) and Root Mean Square Error (RMSE) between estimates at a country level, using only countries where there is complete coverage in both RS1 and RS2. Country-level data available in Additional file 2: Table S2.

While the differences in total biomass for some countries are still very significant, for the majority the two maps agree very well: the mean absolute percentage difference between the two estimates is 12.6%, and the median 8.7%. It seems that the large differences seen in some vegetation classes tend to average out to a certain extent across a country.

#### Reasons for differences between the biomass maps

There are many potential explanations for the differences between these maps, but we here highlight the five we believe are the most likely to be responsible:

i) The lower estimates found on average in *RS1* over *RS2* are most likely to be caused by the different allometric equations used to estimate biomass from the ground plots. Though the equations used in both studies came from the same study [18], *RS1* used the 3-parameter models involving height as well as diameter and wood density, where *RS2* used the 2-parameter models involving diameter and wood density only. Using a non-varying diameter to tree height allometry has been shown to cause a 10-20% overestimate in total biomass, [22]. This also explains the continental differences, as the overestimation using a 2-parameter equation should be strongest in South America, which has the shortest trees, and weakest or reversed in SE Asia, which has the tallest trees; this is exactly what is seen in our comparison (Table 1). The average biomass estimates for the 3-parameter model are about 66 Mg ha^**-1**^ lower than the 2-parameter model over intact Amazonian forest, approximately the same magnitude observed in differences between the two maps in various regions of Amazonia [22]. Although the allometry may introduce a bias between the two maps, the magnitude of bias will have spatial patterns depending on forest types and regional differences in forest structure and allometry [22].

ii) The methodology used in processing and filtering the GLAS LiDAR data may have caused some differences in the height values used in training the spatial modelling of biomass. In both cases GLAS data were filtered if they differed significantly from the SRTM dataset, but only in *RS1* were the data filtered based on slope and signal-to-noise ratio. In both cases pixels were only used for training if at least five GLAS footprints were located within them, and the AGB values from the GLAS footprints were averaged (except for *RS2* in SE Asia, where the criteria was relaxed to greater than or equal to three footprints); this averaging process will reduce noise and to a certain extent smooth out differences in processing, but residual biases from this process could be carried through into the maps.

iii) Different data layers were used to extrapolate the two datasets. *RS1* used QUIKSCAT radar data in addition to layers similar to those used in *RS2*, whereas *RS2* was driven primarily by MODIS and topography data. Equally *RS2* used bidirectionally corrected reflectance (BRDF), EVI2, NII2 and Land Surface Temperate MODIS layers, whereas *RS1* used the seasonal LAI and NDVI MODIS layers. These layers contain different spatial information, and thus despite the use of similar GLAS data, it is likely that these differences changed the spatial patterns in the derived products. Note that none of the data layers used to capture the variations of forest biomass are sensitive to the range of biomass values found in tropical forests and often saturate at low biomass values.

iv) Different modelling environments were used to extrapolate the LiDAR-derived training data: *Maxent* in *RS1*, and *Random Forest* in *RS2*. *Random Forest* is widely used across a wide range of fields for classification and regression, and its bias and error characteristics are well understood [23,24]. *Maxent* is also widely used, especially for classification and species distribution modelling [25], though it is less commonly used, and therefore less well understood, for modelling continuous variables such as AGB. It is likely that this choice of algorithm explains some of the differences in spatial patterns. Both models are considered nonparametric and depend strongly on the statistical approach that optimizes the extrapolation of the training data when the sensitivity of image data layers to biomass is low. In general, *Random Forest* performs better in capturing the mean statistics of the training data, but may suffer from overfitting the training data: as a sign of this *Random Forest* tends to produce considerably higher accuracies against training than test data. *Maxent*, on the other hand, works with probabilities of estimating a class of biomass range, and thus does not necessarily produce a result with a similar mean to the input data, but should produce predictions without overfitting. This leads to *Maxent* producing estimates with similar errors in both training and independent test data, though these errors may be large. In the absence of any global satellite observation of forest structure and biomass all extrapolations will be a compromise between accuracy and overfitting, and only more independent verification datasets will allow for selection of the 'best’ model.

v) Due to mixed input layers neither map is truly a single date product, nevertheless dates of the two maps differ: *RS1* is dated 'early 2000’s’, and *RS2* '2007’. There has been significant land use change across the tropics over this period [3], so it is possible that some of the differences seen could be due to land-use change. However, this cannot explain the large differences in relatively undisturbed areas, for example central Amazonia, nor the many areas where *RS1* is greater than *RS2.*

vi) Some additional differences could be due to the different pixel sizes used: 463 m (*RS2*) vs. 1 km (*RS1*). Larger pixel sizes result in a smaller range of biomass values, due to spatial averaging, and the exclusion of very high biomass values due to landscape heterogeneity. This difference should be especially apparent in the histogram comparisons: RS2 should have a wider distribution than RS1, all else being equal, simply because its input pixel size is a quarter of that of RS1. We performed the analysis at the higher resolution, that of RS2, in order to avoid introducing artefacts by changing pixel values in either dataset. However, as a test, we also reduced the resolution of RS2 to that of RS1 and produced histograms to see if this could be part of the cause of the difference. The histogram results were nearly identical, with the size of every bar within 2% of the size at full resolution, so while resolution could be a factor in the differences observed, it is not the main cause.

### Comparison with FAO 2010 *Forest Resource Assessment*

There is less convergence when comparing the *RS1* and *RS2* maps to the FRA 2010 estimates than to each other (Figure 4c-d, Table 2, Additional file 2: Table S2). The RMSE values for the comparison of the mean country totals of each map with the corresponding values from the FRA dataset are 2–3 times higher than the comparisons directly between the two maps (Table 2). This is not surprising given the very different methodologies used, and the limited capacity of many tropical countries to perform such assessments [26]. However, there is still a significant positive relationship for the mean estimates, and the country totals are remarkably close, particularly for large countries (Figure 4c).

In general the remote sensing maps estimate higher mean AGB values than the FAO values. This is surprising, as the FAO values are reported for forest areas only (the FAO forest definition includes lands with >10% crown cover and also includes plantations), whereas the estimates based on *RS1* and *RS2* include all land, including that not officially classed as 'forest’. The exception to this is Africa where in general FRA 2010 estimates are higher than either *RS1* or *RS2* (Table 2, Additional file 2: Table S2). This is probably due to the larger proportion of non-forest vegetation in these countries, which brings down the average for the *RS* layers but is ignored by the FRA 2010. This is supported by lack of bias in the total country stocks.

### Comparison with a high resolution airborne LiDAR map of Colombia

**Figure 5:**
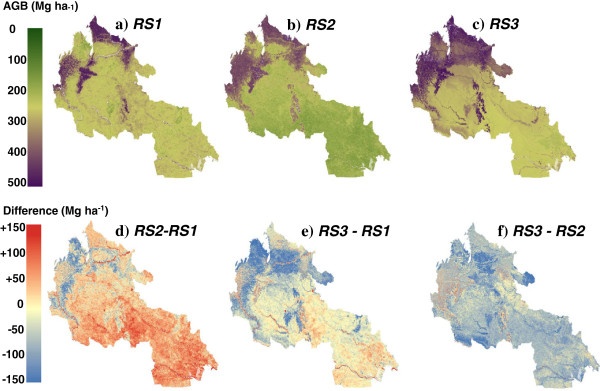
**A comparison of aboveground biomass (AGB) in three remote sensing maps and differences between them for the region of Amazonian Colombia covered by *RS3 *** [21]**: a) *RS1*, b) *RS2*, c) *RS3*, d) *RS2 *– *RS1*, e) *RS3 *– *RS1*, f) *RS3 *– *RS2*.**

We compared the pantropical RS maps (*RS1* and *RS2*) to a recently published AGB map of 165,000 km^2^ of Colombia (*RS3*), derived from field-plot calibrated aircraft LiDAR for 2.8% of the area extrapolated to the region through stratification using optical satellite data, historical forest-change data, and a digital terrain model [21]. *RS3* is expected to have high accuracy (±28% for any given 1 ha pixel) due to its reliance on locally-calibrated high resolution LiDAR data. There are large differences visible between the maps when compared visually (Figure 5), though the broad distribution of biomass is preserved: *RS3* has lower estimates throughout the region, and in particular much lower in the higher elevation areas in the north. The total aboveground carbon stocks differ considerably: *RS1* estimates stocks 23% higher than *RS3*, and *RS2* 42% higher (Table 3)*.*

**Table 3:** Mean and total biomass stocks for a 16 million hectare subset of the Colombian Amazon

**Layer**	**Biomass stocks**	
	**Mean (Mg ha**^**-1**^**)**	**Total (PgC)**
***RS1*** [15]	218.1	1.806
***RS2*** [16]	252.0	2.087
***RS3*** [21]	183.4	1.473

Comparison of the mean and total biomass stocks for a 16,561,695 ha area of the Colombian Amazon mapped in detail at high resolution (100 m) by RS3.

**Figure 6:**
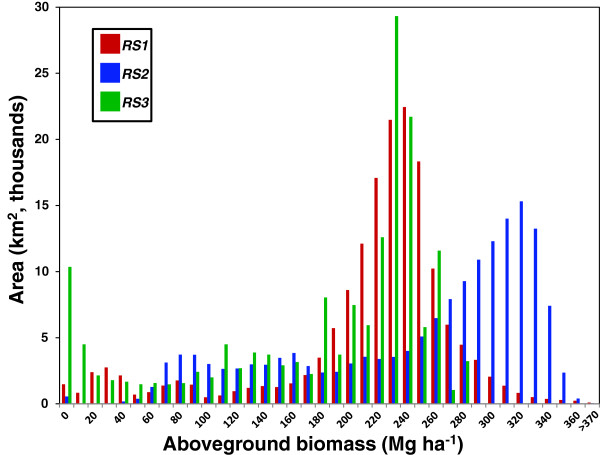
**Histogram showing the aboveground biomass AGB distribution of RS1, RS2 and RS3 over the region of Amazonian Colombia covered by RS3, with 10 Mg ha**^**-1**^**bins.**

When comparing the histograms (Figure 6) a more complex picture appears. There appears to be a very close match between *RS1* and *RS3*, with the high biomass peak for *RS2* offset approximately 90 Mg ha^-1^ to the right (similar to Figure 2a comparing *RS1* and *RS2* for Latin America). However both *RS1* and *RS2* extend to higher values than *RS3*: the highest value for *RS3* is 283.3 Mg ha^-1^, whereas it is 435.7 Mg ha^-1^ and 387.0 Mg ha^-1^ for *RS1* and *RS2* respectively. It is this lack of high values and low estimates in the mountainous regions that explain the low total carbon stock value for *RS3*.

These biomass differences can be explained by a combination of six different factors.

i) *RS3* uses the same allometry as *RS1,* whereas *RS2* uses an allometry excluding height that results in an overestimate of total AGB by 10-20% [22].

ii) Wood density: *RS3* uses local wood density, whereas *RS1* effectively uses South America mean wood density information (contained within its continental Lorey’s height to AGB relationship), and *RS2* uses a mean wood density information across the tropics, contained within the allometries in training data used to develop its pantropical GLAS to AGB relationship. Thus the lower AGB values in *RS3* could be due to especially low wood density in this area.

iii) The relationship between tree diameter and height varies with elevation, soil fertility and geographic location: all three maps treat DBH-height equations differently, with effectively a single equation used for all of South America in *RS1* due to the use of a single Lorey’s height to AGB equation, a single equation for the whole tropics in *RS2* due to the use of an allometric equation that does not include height, and a locally-derived equation for *RS3*. If trees in this region are comparatively short for their diameter, as suggested by the data in [21], then that would explain the lower AGB estimate for *RS3* compared to the other datasets.

iv) Different dates: there may have been significant deforestation in between the creation of the pantropical maps, which have nominal dates of 'early 2000’s’ (*RS1*) and approximately 2007 (*RS2*), and the *RS3* acquisition in 2011.

v) The different resolutions of the three studies, in particular the much higher 100 m resolution of *RS3*, could be influencing the results. It is known that forest biomass scales in a complex manner with resolution, even in a non-heterogeneous landscape [27].

vi) Errors in the extrapolation procedure between LiDAR flight paths and the wider region in *RS3*, in particular the prediction of low biomass values at high elevation areas in the north, and the lack of high biomass values in the densest forest areas, could be erroneous. This final possibility is supported as an alternative map produced in the same study using the same input data but a different spatial extrapolation technique (regression with elevation and the fractional cover of photosynthetic vegetation, rather than a stratification with the same variables plus vegetation history and terrain ruggedness) predicts much higher biomass values in the northern, high elevation areas; and that the field data used to calibrate the LiDAR regression equation has plots with biomass values above 300 Mg ha^-1^, but no pixels in the resulting map exceed 283.3 Mg ha^-1^.

Thus though *RS3* provides an independent test of the pantropical maps, and the comparison is interesting, there are too many uncertainties involved for it to provide validation of one map over the other.

## Conclusions

We found that *RS1* and *RS2* differ significantly in their AGB estimates over a wide variety of forest cover types and scales; however at country level there is general agreement, with much of the country-level difference explained by the choice of different allometric equations. This has an important implication for REDD+ — it appears we have the algorithms and tools to estimate biomass stocks with some certainty, and the largest uncertainties in setting up deforestation baselines relate to forest cover changes (rates of deforestation/degradation) [3,28].

When summed to a regional scale, *RS2* estimates on average higher biomass values than *RS1*. This is almost certainly due to the different choice of allometric equations, with the 2-parameter equations excluding height used in *RS2* known to consistently estimate higher biomass values than the 3-parameter equations including height used by *RS1*[22]. Further differences between the layers could be due to a variety of factors, including their different ground and remote sensing input data, different modelling environments, and different pixel sizes. It is also clear from comparison to a high resolution, locally calibrated map (*RS3*) that a further limitation present in both studies is the lack of local wood density or diameter-height calibration. Both are known to vary considerably across the landscape [22,29] but the use of a single (*RS2*) or three continental (*RS1*) equations relating GLAS LiDAR footprints to AGB smooths out these variations.

All three remote sensing maps compared here actually use a very similar processing chain to produce their AGB maps, despite the difference in scale and resolution between the pantropical maps (*RS1* and *RS2*) and the regional map (*RS3*). They all use LiDAR data to produce distributed estimates of canopy height (ICESat GLAS for *RS1* and *RS2*, aircraft LiDAR for *RS3*), convert these to AGB using field data located under the LiDAR footprints and generic allometric equations, and then use these points to train model biomass across the landscape using ancillary data layers, including optical satellite data and terrain information. This method makes intrinsic sense, balancing the cost to accuracy trade-off of field, LiDAR and optical data, and should produce internally consistent products that can be validated against independent field datasets. Such a processing chain could be followed by most projects attempting to create baseline carbon maps, and adapted to reflect existing input data available, and the required accuracy. There has been little work as yet on the uncertainties associated with differencing products produced in this way for different years to assess changes due to deforestation, degradation, and forest growth: as REDD+ payments are effectively based on differences in carbon stocks, it is important that further work is done in this area.

Quantifying emissions from deforestation has largely made use of simple book-keeping models based around FAO and IPCC data [1,30], and more recently explicit carbon maps to quantify stocks before deforestation at a pixel level [3]. The results here support the latter approach: it is clear that carbon stocks vary greatly within the forests of every country, and that is important because deforestation within a country is also not evenly distributed. For this reason information on the spatial distribution of stocks would be expected to improve upon estimates based strictly on sampling approaches.

Currently the carbon stocks for a region or country are often based on guideline mean biomass values for particular vegetation types [31] or on country-specific mean carbon stock values, for example from FRA 2010 [20]. These results suggest that pantropical biomass maps can provide much better estimates of carbon stocks at a project or national level, and despite some differences, independent maps show a high level of consistency. We hope that these products, and improvements on them, are widely used. All three maps compared here contain detailed error propagation procedures, and give confidence intervals at both a pixel and regional level [15,16,21]. Ultimately the only way to truly quantify the errors on biomass maps of these scales would be to perform the destructive harvest of plots the size of a whole pixel, which is impractical, so these uncertainty estimates are themselves only estimates of the true error. However, error propagation methods for biomass mapping are now well established [9,32], and the relative agreement between all three independent maps, at least at a regional scale, provides some confidence in this procedure.

Despite the general agreement discussed above, we cannot ignore the large differences between the maps in some areas (Figure 1). These tend to be areas where we have the least field data, most notably in central Amazonia, the Congo basin, and Papua New Guinea. Field campaigns, ideally combined with destructive tree harvesting to reduce uncertainties in allometries, and airborne LiDAR to allow for accurate spatial extrapolation across a landscape, would be particularly useful to improve our understanding of the carbon stocks in these regions.

### Data preparation & methods

We performed all re-projections and subsequent analyses of remote sensing data using IDL-ENVI 4.8 (Exelis VIS), and all area summation calculations using ArcMap 9.3.1 (ESRI). The original AGB datasets (*RS1*[15] and *RS2*[16]) were provided by the authors in their native projections and resolutions: 0.00833 degrees (c. 1 km) and a geographic (WGS-84) projection for *RS1*, and 463 m and the MODIS sinusoidal projection for *RS2*. In order to avoid introducing artefacts by changing the true resolution of either dataset or averaging any pixel values, we warped *RS1* to the same projection and resolution as *RS2*, using a rigorous arithmetic conversion between the projections and a nearest neighbour resampling method (so no pixel values were changed). This had the added advantage that the subsequent analyses all took place in an equal area projection (sinusoidal), simplifying area-summation and averaging calculations. *RS3*[21] was provided in a Universal Transfer Mercator (UTM) projection at 100 m resolution; we reprojected it to the 463 m MODIS sinusoidal projection of *RS2* using a rigorous transformation and cubic convolution for comparative figures, and left it at its native resolution for summation calculations. *RS3* was provided in units of Mg C ha^-1^, so we converted it to Mg ha^-1^ (dry biomass), the same units as *RS1* and *RS2*, by dividing by 0.485, the conversion stated in the paper [21].

We used two vector datasets to subset the AGB maps in different ways. First we queried the data using country outlines from the ESRI Data & Maps Database, using the World Countries layer updated on 17^th^ January 2012. Second we used the Global Land Cover 2000 (GLC-2000) as a vegetation cover dataset [33]; this dataset has been shown to be globally consistent [34], uses a biologically-relevant hierarchical legend based on the FAO Land Cover Classification System, and was used as a core dataset in the Millennium Ecosystem Assessment. Its 1 km resolution is comparable to the remote sensing datasets.

We compared the different raster layers directly, and through comparison of averages within the vector datasets. We also compared the datasets at a country level to the total carbon estimates from the FAO’s 2010 Forest Resource Assessment (FRA) [20]. In all cases we converted dry biomass (the units of *RS1* and *RS2*) to carbon (the units of the FRA) by multiplying by 0.5 (following that used by *RS1* and *RS2*, but differing from the 0.485 used originally in *RS3*), and carbon to tCO_2_e by multiplying by 3.667 [9].

### Data

The datasets used in this study have been made available by the authors. *RS1* is available at http://carbon.jpl.nasa.gov/data/dataMain.cfm, and *RS2* at http://www.whrc.org/mapping/pantropical/carbon_dataset.html. Additionally the three datasets can be compared interactively at http://carbonmaps.ourecosystem.com.

## Competing interests

The authors declare that they have no competing interests.

## Authors’ contributions

The study was devised by EM, SS & SG. EM performed the analysis and produced the figures using data layers provided by SS, AB and GA. EM wrote the text, with substantial contributions and edits made by all other authors. All authors read and approved the final manuscript.

## Supplementary Material

Additional file 1 **A comparison of the mean aboveground biomass (AGB, Mg ha**^**-1**^**) in different landcover classes from the Global Land Cover 2000 map between *RS1***[15]** and *RS2***[16]** by continent and across the tropics.** Water bodies are excluded from these calculations. Click here for file

Additional file 2 **A comparison of the mean, median, maximum and total carbon stock by country in three datasets: *RS1***[15]**,*RS2***[16]** and the FAO Forest Resource Assessment (FRA) 2010.** The total area of the country within the RS maps is also included: where this is different to the total area of the country the figures are put in italics, and comparisons with the FRA data (which are for the full country) are not valid. Countries have only been included if greater than 50% of their surface is covered by the RS maps. Water bodies are excluded from these calculations. Click here for file
